# Self-managing individual wellness for the health professional: A somatology perspective

**DOI:** 10.4102/hsag.v24i0.1119

**Published:** 2019-05-29

**Authors:** Karien Henrico, Jeanette E. Maritz, Johan Bezuidenhout

**Affiliations:** 1 Department of Somatology, University of Johannesburg, Johannesburg, South Africa; 2 Department of Health Studies, University of South Africa, Pretoria, South Africa; 3 Division Health Sciences Education, University of the Free State, Bloemfontein, South Africa

**Keywords:** individual wellness, health professional, self-management, somatology, personal wellness, healthcare provider, demanding work environment, wellness

## Abstract

**Background:**

Health professionals play a vital role in the stability and sustainability of any healthcare system. However, the well-documented long working hours, lack of wellness support structures, regular occurrence of burnout and low retention rates are concerning.

**Aim:**

The aim of this research study was to understand how a group of therapists self-manage their own individual wellness, to provide insight on how other health professionals, working in a demanding environment, could potentially address their individual wellness more effectively.

**Setting:**

The research was conducted in a private room at the place of participant employment, in two metropolitans in Gauteng, South Africa.

**Method:**

Qualitative, explorative, descriptive and contextual designs were used within the paradigm of constructivism. Purposive sampling was used to select participants. Data were collected through in-depth interviews, field notes and reflective practices, and analysed through open coding.

**Results:**

This study revealed disequilibrium between the theoretical knowledge and practical realities of therapists, and indicated that these therapists experience various personal obstacles that hinder the self-management of their individual wellness.

**Conclusions:**

Somatology therapists use various personal strategies that allow them to better self-manage their individual wellness. Individual wellness seems to be a personal phenomenon, indicating the need to self-reflect on personal perceptions of wellness, individual wellness obstacles and individual wellness strategies to effectively self-manage individual wellness.

## Introduction and background

The sustainability and quality of any healthcare system relies heavily on the health professionals who provide the care (MacDonald et al. 2011:18). The continuous growth of the healthcare resulted in heavy workloads for individuals working within this demanding sphere (MacDonald et al. 2011:18). A study by Cho, Laschinger and Wong (2006:1) found that many health professionals leave their jobs within 2 years of graduation and have symptoms of burnout, depression and emotional exhaustion. Another study found a connection between burnout and increased suicidal tendencies in medical students (Dyrbye & Shanafelt 2011:801). Not only did these studies highlight various concerns for the health professionals themselves, but also the negative impact on patient care and satisfaction (Richter & Jooste 2013:5). The demands faced by health professionals are often recognised as researchers seek to develop interventions aimed at improving health professionals’ wellness (LeBlanc, McConnell & Monteiro 2015:265). This study explores how somatology therapists self-manage their individual wellness, and thus aims to provide insight on how other health professionals could possibly address their individual wellness needs more effectively.

### Individual wellness

Collectively, wellness and wellness interventions have received increasing attention from researchers in various spheres (Horton & Snyder 2009:216). This might be because of the fact that wellness is perceived to enhance a sense of well-being in individuals who are not ill (McGrady et al. 2012:254) and the fact that wellness promises increased productivity (Conner 2013:63). Because of the popularity of wellness-related literature, the term wellness has rapidly changed over time. Wellness can be defined as a holistic, multi-dimensional, active way of life through which individuals become aware of and make choices for optimal health and well-being (McGrady et al. 2012:255).

Wellness is only considered valuable once it is understood and integrated by an individual (Horton & Snyder 2009:216). Consequently, the notion of individual wellness has grown substantially over the past 20 years. Individual wellness refers to the emotional, physical, spiritual, intellectual, social, environmental and interpersonal well-being of an individual (Myers & Sweeney 2004:234). The benefits of addressing individual wellness extend beyond the individual’s personal gain, as clients, family, friends and others can also be positively affected.

Wellness interventions often focus on self-efficacy (Lockwood & Wohl 2012:628), self-care and team building (Zadeh, Gamba & Hudson 2012:295), physical and psychosocial functioning, counteracting negative thoughts and adaptive coping (Dorough et al. 2014:3). Although these studies have proven beneficial in their focal area, wellness scholars suggest that addressing wellness needs to focus on much more than these isolated variables (Myers & Sweeney 2008:482). Simply acquiring knowledge about wellness behaviour does not lead to behavioural change; there should be a desire to change (or control) behaviour once the individual is made aware of wellness hindering behaviour (Horton & Snyder 2009:222), hence the strong focus on self-management within the current study.

### Self-management of individual wellness

Self-management has become a common term for various behavioural interventions and several healthy behaviours (Lorig & Holman 2003:1). Research on self-management is focused on health disorder management (such as diabetes, spinal injuries, mental health and chronic illness) and implementing self-management in educational settings, particularly pertaining to problematic children in classroom settings (Omisakin & Ncama 2011:1734). Self-management seems to mean different things in different fields (Briesch & Daniels 2013:108). For the purpose of this study, self-management relates to one’s own perception and behaviour when dealing with cognitive and emotional processes and consequences, which includes the individual’s body, mind and spirit, as they become aware of the self and their adaptive capabilities (Özkan 2011:809). There seems to be consensus among researchers that the term self-management can be applied to health-promotion initiatives such as wellness (Omisakin & Ncama 2011:1733).

## Problem statement and aim of the study

As wellness professionals, somatology therapists are tasked with guiding their clients on various topics pertaining to their health and well-being (Boshoff 2012:93). The Global Spa Summit (2010:iii) emphasised that the somatology scope of practice belongs to, and often overlaps with, the wellness cluster that focuses on improving quality of life in the area of complementary and alternative medicine, spa, healthy eating and weight loss, wellness tourism, personal health, workplace wellness, fitness and anti-aging industries, to mention but a few. The current literature suggests that somatology is interwoven throughout the therapies mentioned and plays a fundamental, often disregarded, role in all aspects of wellness and well-being tendencies, especially in women but also in men (Richter 2010:50). Somatology training includes theoretical wellness concepts and practically cultivating such concepts into the well-being of clients, especially in the sense of ill-health prevention (Straughan 2010:468). Because of the nature of the industry and their professional wellness expertise, it is expected that somatology therapists would apply their theoretical knowledge to their own individual wellness practices. This is not always the case, as with many other health professionals.

Research has not yet attempted to understand how somatology therapists foster their own individual wellness within their demanding, ever-expanding industry riddled with various wellness challenges (Campbell 2012:11). By sharing the understanding of how somatology therapists self-manage their own individual wellness, this article aims to give insight on how other health professionals who work in demanding environments might be able to better address individual wellness.

## Research design and methods

Qualitative (McKenney & Reeves 2013:74), explorative, descriptive and contextual designs were used in this study, within the paradigm of constructivism that allowed each participant to actively unfold their own realities (Cooperrider 2008:23). This design is best suited for this study, as the lived experiences of the participants were important to address the research problem (Richter & Jooste 2013:9).

### Population and sampling

The target population was composed of somatology therapists practising in two metropolitans in Gauteng, South Africa. Purposive sampling was used to select information-rich participants (Marshall & Rossman 2011:111), as proportionality was not the primary concern of the study. Potential participants were sourced through the use of Google™, by advertising in local newspapers and magazines, and sourced from graduates from two metropolitan universities. The sampling criteria included the following:

- therapists practising for 3 years or longer
- therapists with at least a National Diploma in Somatology, or equivalent qualification
- therapists proficient in English.

Possible participants were contacted via telephone to invite participation. An appointment was booked for the researcher to meet the participant at a suitable time, once they volunteered to partake in the study. A total of eight therapists participated in this study, whereafter data saturation was achieved.

### Data collection

After volunteering to partake in this study, each participant received an information sheet and consent form, providing them with detailed information about the study. In-depth interviews were conducted by the researcher in an area where the participants felt most comfortable (mainly their own treatment room). Participants were asked open-ended questions regarding their current reality of self-managing their individual wellness, what obstacles they faced in self-managing their individual wellness and what strategies they use to overcome these obstacles. Probing was used to prompt responses and explore certain topics in greater detail (Okun & Kantrowitz 2014:76). All interviews were digitally recorded and took approximately 30 min. Field notes, critical to qualitative studies, described the detail of the people, places, things and events (Given 2008:342). This allowed the researcher to record what was done, seen, heard and felt during the interviews and the time spent with the participants. Furthermore, a reflective journal was kept by the researcher to address the methodological, ethical and emotional challenges that arise when conducting qualitative research (Rolls & Relf 2006:290). This allows the researcher to examine and explain, as unambiguously as possible, ways in which they might have influenced the research.

### Data analysis

Data were analysed by means of open coding (Creswell 2013:192). The qualitative data analysis allowed the researcher to explore and debate various aspects that are important to the somatology therapist as pertaining to how individual wellness is self-managed. For this study, the researcher used the seven steps of Tesch’s data analysis procedure, as described by Creswell (2013:109):

1. An overview of the entire picture was gained by carefully reading through the verbatim transcriptions. Ideas that surfaced were written down.
2. The researcher chose the first interview of the study and read through it asking ‘what is it about?’ – not reading for information but rather the essential meaning of the data. The researcher wrote her thoughts in the margin of the page.
3. Once the researcher completed all the interviews mentioned earlier, she compiled a topic list. Similar topics were grouped together and placed into columns named ‘major topics’, ‘unique topics’ and the ‘leftover topics’.
4. Then, the researcher returned to the data with this list. Each topic was abbreviated as a code and the codes were written next to the appropriate segments of the text. The researcher tried out this preliminary consolidating scheme, to see whether new categories and codes emerged.
5. Thereafter, the researcher looked for a descriptive wording for the topics and grouped them into categories. The total list of categories was then further reduced, by once again grouping similar topics.
6. A final decision was made on the abbreviations for each category and the codes were captured on a separate sheet.
7. The verbatim quotes in each category were listed under the appropriate heading and used as evidence.

A set of unmarked transcripts was given to an independent coder with experience in qualitative data analysis, who then replicated the steps. The researcher and the coder met for a consensus discussion.

### Trustworthiness

Trustworthiness was maintained by using Lincoln and Guba’s (Creswell 2013:203) model of criteria that include credibility, transferability, dependability and conformability with the inclusion of authenticity (Tobin & Begley 2004:132). Credibility was ensured by prolonged engagement, triangulation, using peer review and reflexivity. To ensure that this study is transferable to other research contexts, thick descriptions of research methods and purposive sampling were used. Dependability was ensured by the code–recode procedure and by describing the research methodology. Confirmability was ensured by using an independent coder and having a chain of evidence. Enlarging personal constructs, appreciating the views of others, stimulating some form of action and empowering participants allowed for authenticity within this study.

### Ethical considerations

The ethical considerations that were adhered to in this study included right to privacy, right to autonomy and confidentiality, right to fair treatment, right to protection from discomfort and harm and obtaining informed consent (Grove, Burns & Gray 2012:193). Participants could choose to participate in this study or not, were informed of their right to withdraw during the study, no personal information was shared with other parties, participants were treated with integrity and respect and participants were asked to sign a consent form before participating in the interview. Ethical approval was granted by the University of Free State [ECUFS Nr. 45/2013].

## Findings and discussion

Three main categories and several sub-categories were identified. A summary of all the findings is presented in Table 1.

**TABLE 1:** Categories and sub-categories that emerged from the analysis.

Categories	Sub-categories
1. Individual wellness disequilibrium in the current reality of the therapist	1.1. Insufficient self-care 1.2. Emotional guilt 1.3. Cognitive disequilibrium
2. Personal obstacles that hinder the self-management of individual wellness	2.1. Physical obstacles 2.2. Emotional obstacles 2.3. Cognitive obstacles
3. Personal strategies to overcome self-management obstacles	3.1. Physical self-care 3.2. Emotional self-care 3.3. Cognitive self-care

### Individual wellness disequilibrium

The findings indicated a wellness disequilibrium in terms of the theoretical knowledge and what is practised in relation to individual wellness. Participants expressed a lack of self-care, feelings of emotional guilt and cognitive-related difficulties. It can be assumed that, as a specialist in the field, somatology therapists understand each of these topics, but self-managing themselves daily seems problematic.

#### Insufficient self-care

The data from the present study clearly state that all participants were fully aware of the importance of self-care and knew what it entails, yet expressed an inability to take care of the self.

‘…that burned me out, in the sense that nothing fills your tank, because all those things keep you busy the whole time, you give to people the whole time. And I realise that it is important to have something that fills you tank. And I enjoyed sport, I always played hockey and well, I do not get time and so …’ (Participant 6, female, somatologist)^1^

Myers and Sweeney (2008:485) stated that self-care is taking responsibility for your own wellness, self-attention and safety habits that are pre-emptive in nature. Self-care forms part of the essential self (Hattie, Myers & Sweeney 2004), hence is concerned with taking accountability for one’s own wellness, and mainly involves preventative behaviour as well as remedial treatment. If an individual lacks a sense of well-being, such as feeling tired, he or she tends to be less enthusiastic about self-care (Hattie et al. 2004). Therefore, it is no surprise that not having the time for adequate self-care could lead to guilt, especially for a professional working in such a field.

#### Emotional guilt

Many of the therapists expressed a sense of guilt towards their ability to self-manage individual wellness, as indicated in the way Participant 7 shook her head while saying the following:

‘I have a small baby, so it is actually hard to juggle work, myself, the house… and so there, I would say yes… that is actually one of my weaknesses, to be able to just, you know, what can I say it’s work, life and being able to actually manage.’ (Participant 7, female, somatologist)

The people-orientated nature of working in the health professional arena is personally demanding, lacking time to rest, relax, recharge and spend quality time with loved ones (Boshoff 2012:56), especially for female health professionals. The literature clearly indicates that women often put the needs of others before their own in their quest to balance the multiple roles in their family, society, home and career environments (Omisakin & Ncama 2011:1734), leading to escalated feelings of emotional guilt.

#### Cognitive disequilibrium

Participants reported that they felt they had insufficient skills to plan effectively, with emotions being the main cause of interference.

‘I am very quick to let my emotions get in the way of making a decision.’ (Participant 2, female, somatologist)

Lack of time management skills was mentioned as adding pressure to the therapist’s life. Therapists indicated the importance of time management when considering self-management and individual wellness, for example by making sure to avoid double bookings and overloading the schedule:

‘So I think that time management is important for me at this stage… it is important and I need to look at it.’ (Participant 3, female, somatologist)

Self-reflection evolved as an important aspect of self-managing individual wellness. During the interviews, therapists reflected on their own wellness disequilibrium and what they perceived would improve their own self-management and individual wellness.

### Personal obstacles that hinder self-management

During data analysis, physical, emotional and cognitive obstacles that might hinder the individual to self-manage their own wellness were revealed.

#### Physical obstacles

When looking at wellness literature (Horton & Snyder 2009:219), it came as no surprise that in this study physical wellness obstacles are stress and lack of relaxation time. Therapists are aware that stress reduces the ability of the individual to be physically well.

‘Yes, stress is the big thing, because I think if you are not stressed… automatically your physical well-being will be better.’ (Participant 1, female, somatologist)

The therapists in the present study acknowledged the importance of making time for physical well-being, but this is hindered by a lack of available time. In the following example, the participant expressed her desire to pursue a hobby she enjoyed, to assist with stress-related symptoms and to foster relaxation, yet she does not have the time to do so.

‘…and that started to burn me out, in the sense that nothing fills your tank, because all those things keep you busy the whole time, you give to people the whole time. And I realise that it is important to have something that fills your tank. And I enjoyed sport, I always played hockey and well, I do not get time and so…’ (Participant 2, female, somatologist)

Taking care of the physical self is vital for avoiding burnout (Richter 2010:6), and the nature of self-care stresses the importance of sufficient and appropriate attention to the self (De Jong 2010:205). The present study confirms that self-care is needed to allow for individual competence and judgement to not become impaired, that is, they should ‘walk their talk’ (De Jong 2010:204).

#### Emotional obstacles

Participants identified emotional obstacles to be feelings of guilt and violation of emotional boundaries. Guilt was expressed in terms of being a team leader by Participant 2. She shared the notion of the client always being right, therefore taking the side of the client rather than the therapist when there was a problem with a treatment offered. The following day, after self-reflection, she realised the situation was not dealt with correctly resulting in feelings of guilt towards her team member as seen in the following statement.

‘…and at the end of the day you realise it was really not right.’ (Participant 2, female, somatologist)

This emphasises the importance of the trial-and-error stage of self-management, as discussed by various authors (Gerhardt 2007:12; Richter 2010:4).

Not having emotional boundaries seems to add pressure on therapists and hinders their ability to self-manage their wellness. Participant 3 expressed how she gets emotionally involved with client-related issues.

‘I very quickly get emotionally involved.’ (Participant 3, female, somatologist)

Many therapists are unaware of the emotional labour required of the somatology therapists (Richter & Jooste 2013:1). A succession of clients with varying levels of energy, well-being or communication patterns can be challenging for therapists, who must rapidly adjust to different styles and patterns, if they do become emotionally engaged. Transitioning between emotional levels is often problematic.

‘And it is sometimes difficult if you get a depressed client, to get to her level and say, you know it’s not really that hectic, and then the next client is happy and you need to get to that level again, so to balance it is sometimes a challenge.’ (Participant 4, female, somatologist)

‘…in our industry you have to emotionally be on the client’s level the whole time.’ (Participant 5, female, somatologist)

Health professionals, working in people-orientated jobs, need to create emotional boundaries as a strategy to advance self-management and individual wellness (Ekendahl & Wengstrom 2008:43) and help to deal with work-related stressors. Developing emotional boundaries can also include incorporative coping strategies to allow the individual to disengage from the previous client and focus care on the current treatment (Ekendahl & Wengstrom 2008:45).

#### Cognitive obstacles

The present study revealed that self-management is often hindered by a lack of time management and managerial skills. Time management is often linked to self-regulation (Briesch & Daniels 2013:72). Time management seemed to be a general problem for managers and therapists, as it was mentioned by most of the participants.

‘…I realise that time-management is a big thing for me at this stage, because there are many things that need to happen.’ (Participant 2, female, somatologist)

Because of the long working hours, generally associated with being a health professional, optimising the limited time available is vital. As long working hours is a clear obstacle in one’s self-management capabilities.

‘Obstacles… it will be the hours of the industry… overworking yourself and feeling tired.’ (Participant 4, female, somatologist)

Participants also indicated that a lack of managerial skills, in terms of delegation and planning, hindered their self-management.

‘I would have to learn to delegate, to trust people to do things their way even though it is not my way.’ (Participant 2, female, somatologist)

Cognition in terms of self-management strategies and obstacles is complex. We are of the opinion that self-knowledge and self-refection will allow the individual therapist to better deal with cognitive obstacles in their self-management and individual wellness journey.

### Personal strategies to overcome self-management obstacles

Participants discussed the strategies they used to overcome the self-management obstacles they encountered. Although participants reported disequilibrium between their reality and what they expressed as a state of successful self-management and individual wellness, they had found ways in dealing with the obstacles. As seen below, the application of coping strategies varied for each individual.

#### Physical self-care

As a whole, participants in this study were dissatisfied with their own physical self-care. This may be because of therapists often not caring for the self as much as they expect their clients to care for the self. Physical self involves all ‘biological and physiological processes that compose the physical aspects of a person’s development and functioning’ (Myers & Sweeney 2008:485), so a single strategy will not foster a sense of physical self-care, but a combined effort is needed of various strategies that are important to the individual. Regular treatments and breaks, healthy eating, exercise and relaxation aimed at resting the body were mentioned by therapists in our study as physical self-care strategies.

‘I do my regular waxes, but stuff like facial, mani’s, pedi’s I never get to …I try using the right products on my face, specifically the products that we work with and recommend to clients.’ (Participant 4, female, somatologist)

‘If I could have more time to do like my exercises, like I’m supposed to, and eat the right diet and have my regular massage and mani, pedi and facial, like I know I should be.’ (Participant 3, female, somatologist)

As seen in the quote above, eating a healthy diet could foster a feeling of physical self-care. Exercise and healthy eating are seen to run alongside and influence each other as they form part of the physical self of individual wellness (Myers & Sweeney 2008:485). This is re-emphasised in the following quote:

‘So in terms of all these things… to improve yourself, and then your health also, without exercise, so I know automatically you want to eat more healthily and so physically you will also want to work on changes.’ (Participant 8, female, somatologist)

Lastly, therapists expressed that relaxation (aimed at resting the body) is important within a health professional context.

‘… I think what our hours ne, sometimes they can be…, we are overworked basically sometimes and I think if they could just find a way to try and relax.’ (Participant 4, female, somatologist)

Relaxation might not always be possible, as observed by Participant 6, because of the time constraints and long working hours.

‘…when I’m off I tried to get as much rest as possible and I try to eat right, but it’s not easy due to the hours that we work.’ (Participant 6, female, somatologist)

Physical self-care is important as it has been linked to impaired competence and judgement (De Jong 2010:211). Improving physical self-care leads to a reduction in individual distress and preventing of burnout (Merluzzi et al. 2011:15). By taking care of ourselves first, Richards (2013:199) says that we bring positive energy and vitality that positively affects others and influences all aspects of individual wellness. Therefore, physical self-care should be fundamental to any individual wellness recommendations.

#### Emotional self-care

Emotion is complex, is steered by thought and includes a positive attitude, emotional control and self-awareness (Van der Cingel 2009:124). The participants suggested, for example, a few minutes of silence before bed, debriefing, yoga, exercise and taking time for relaxation, as shown in the following examples.

‘Well just a few minutes at night before bed where I make myself silent for a few minutes … Just to relax, to unwind and to see what happened today, what is going to happen tomorrow, just to recap.’ (Participant 3, female, somatologist)

‘Partake in relaxation or debriefing techniques and … maybe yoga… you do it to improve your stress…’ (Participant 1, female, somatologist)

Participant 2 suggests maintaining emotional self-care by means of a positive attitude, which would automatically lead to improved self-management:

‘…focus on positive aspects and to improve those… you will automatically manage yourself better.’ (Participant 2, female, somatologist)

Staying positive helps to reduce levels of stress, which leads to the successful adaptation to a stressful situation (Baldacchino & Draper 2001:834). A positive, optimistic outlook on life enables the individual to cope in a crisis situation (Baldacchino & Draper 2001:835). It seems important to mentally prepare to stay positive:

‘But also mentally how do you prepare yourself and how do you keep yourself positive, especially when you working with lots of clients all time so mentally… to keep yourself positive throughout.’ (Participant 3, female, somatologist)

The emotional self-care strategies discussed included a sense of keeping emotional control by ‘letting go’ of certain situations or encounters with colleagues and clients.

‘Well I try not to let things get to me, like for instance if … maybe I say I get in an argument with [colleague] and my clients are here, I try to not let it take over because when it does, it prevents … like your whole day basically, so certain things I just try to deal with them like, I just calm myself down basically.’ (Participant 8, female, somatologist)

The ability to ‘let go’ was also discussed in a previous study as being important in order for the therapist to self-manage (Richter 2010:4). If a therapist deals with clients on a daily basis, letting go could seep into various aspects of their ability to self-manage (Richter 2010:8), and this is a process involving mindful choices in using emotional, cognitive and behavioural tactics.

Finally, the therapists discussed self-awareness as a strategy to overcome obstacles hindering self-management. As one participant observed, self-awareness and mental health go hand-in-hand, supporting the view that emotional self-care is concerned with learning to calm emotional distress and deepen self-awareness (Richards 2013:198).

‘Basically managing myself… my self-appearance but also my mental health – how I feel about things, my stress levels…’ (Participant 3, female, somatologist)

To ‘function optimally’, therapists need to experience, understand, regulate and express emotions at a level that facilitates each situation and this skill includes learning to monitor oneself continuously (Langhoff et al. 2008:68). Identifying the emotions is critical in any therapeutic process (Merluzzi et al. 2011:16). It is vital for a therapist’s well-being to receive high levels of emotional support, demonstrating the importance of including emotional strategies in a balanced, self-managed individual wellness programme (Langhoff et al. 2008:69).

#### Cognitive self-care

Participants discussed cognitive self-care as goal-setting, healthy boundaries, self-development, self-reflection, self-discipline and taking up a hobby. Participant 1 mentioned goal-setting as she reflected on her inability to reach a peak of self-management and individual wellness, perhaps because her own goals were not realistic, indicating that reflection is also important in the process of achieving preset ambitions.

‘Set goals and try to reach them and if you do not … look at what is in your way, why is it not working, those things lead to wellness to improve your situation of where you are in life.’ (Participant 1, female, somatologist)

Goal-setting improves:

task performance by focusing and directing the therapist, by regulating their efforts, by enhancing their persistence on a given strategy or task, and by promoting the development of new strategies for improving the therapist’s task performance. (Moore et al. 2001:257)

Another study reinforced the view that goal-setting is fundamental to self-management (Gerhardt 2007:12).

Setting healthy boundaries is an important cognitive self-care strategy, specifically between colleagues and in the therapist–client relationship, even if it needs time to master the skill.

‘…to let them be disrespectful and to think that it’s not necessarily right if you want a place like where you… you will create an atmosphere where there is mutual respect for colleagues as well as clients.’ (Participant 7, female, somatologist)

‘…I have been working here for a long time, so my clients know that if they get here, I do not want their nonsense … [*laugh*].’ (Participant 6, female, somatologist)

In addition, Participant 4 indicated that acquiring methods to self-develop might facilitate self-management: ‘You can attend a course…’ (Participant 4, female, somatologist).

Participants suggested that self-reflection might assist in overcoming perceived self-management obstacles to managing individual wellness. Their comments express the belief that one should be critical of the self and see this as a way to grow. Self-reflection could be used in terms of looking back at the day and asking how things could have been done differently. Participant 2 (female, somatologist) observed that ‘self-reflection is an important thing’ and Participant 4 (female, somatologist) saw the self-reflection approach as being beneficial, ‘…take it’s a self-growth, good criticism I can learn something from it’.

Our study echoes the view that self-care and ongoing self-reflection are crucial for giving care to others, for being personally well and for appreciating the self (Drick 2014:468). Self-reflection should be used not only to see how things could have been done differently, but also as a motivational tool: ‘what was done well during the day?’

All the accounts of self-management and individual wellness strategies mentioned by the participants indicated the need for the therapist to create a balance, supporting numerous previous studies. Ufuk and Ozgen (2001:96), for example, concluded that most female professionals find it difficult to allow sufficient time for the self. This is an area where most conflict might arise because they often give little priority to the self and because women tend to sacrifice their hobbies and desires to balance their household and occupation. A balanced lifestyle allows more time for the self and, in turn, leads to better self-management and individual wellness (Richter 2010:4).

## Conclusion

Understanding how therapists in our study self-manage their individual wellness might provide valuable insight to other health professionals working in demanding, people-orientated jobs. This study demonstrated disequilibrium between the theoretical knowledge and practical realities of therapists, indicating that therapists experience various personal obstacles that hinder the self-management of individual wellness. The therapists used various personal strategies to better self-manage their individual wellness. It was noteworthy that the therapists echoed the views that self-care and ongoing self-reflection aiming at creating some form of balance allowed the individual to support the self-management of individual wellness. Individual wellness seems to be a personal phenomenon, meaning that one person’s wellness needs will differ from another individual, wellness perceptions will be unique to each individual and their road map to achieving wellness will also be as unique as the individual themselves.

Further research is needed to understand how reflecting on various wellness dimensions and self-management skills could influence one’s perception of wellness obtainment. Because of the well-documented benefits of holistically well health professionals, no healthcare worker can risk not addressing their own individual wellness. A similar study can also be conducted in other health professional domains.

### Limitations

Targeting only somatology therapists, who are female, provided a one-sided opinion. Men and other health professional might have other experiences that could benefit this topic. Also, this was a small-scale study confined to Pretoria and Johannesburg, South Africa.

## Footnotes

1 In the interest of authenticity, all quotes are presented verbatim
